# DNA Barcodes Confirm the Taxonomic and Conservation Status of a Species of Tree on the Brink of Extinction in the Pacific

**DOI:** 10.1371/journal.pone.0155118

**Published:** 2016-06-15

**Authors:** Craig M. Costion, W. John Kress, Darren M. Crayn

**Affiliations:** 1 Botany Department, National Museum of Natural History, MRC 166, Smithsonian Institution, Washington, DC, United States of America; 2 Australian Tropical Herbarium, James Cook University, Cairns Campus, Cairns, QLD, Australia; 3 Centre for Tropical Environmental Sustainability Science, James Cook University Cairns Campus, Cairns, QLD, Australia; University of Hong Kong, CHINA

## Abstract

The taxonomic status of a single island, narrow range endemic plant species from Palau, Micronesia (*Timonius salsedoi)* was assessed using DNA barcode markers, additional plastid loci, and morphology in order to verify its conservation status. DNA barcode loci distinguished *T*. *salsedoi* from all other *Timonius* species sampled from Palau, and were supported by sequence data from the *atpB-rbcL* intergenic spacer region. *Timonius salsedoi* was only known from two mature individual trees in 2012. Due to its extremely narrow range and population size, it had previously been recommended to be listed as Critically Endangered Status under three separate IUCN Criteria. In 2014 a second survey of the population following a typhoon revealed that the only two known trees had died suggesting that this species may now be extinct. Comprehensive follow up surveys of suitable habitat for this species are urgently required.

## Introduction

Oceanic island environments are widely recognized to be highly vulnerable to habitat loss, invasive species, and other environmental threats consequently giving them the world’s highest modern species extinction rates. A total of 72% of all recorded extinctions since 1500 AD for mammals, birds, amphibians, reptiles and mollusks occurred on islands [1]. The Pacific Islands, which as a whole are recognized as the Polynesia-Micronesia biodiversity hotspot [2], have been shown to be especially vulnerable with more recorded extinctions occurring there since 1984 then in any other biogeographic region [1]. A wealth of literature indicates that species extinctions became prevalent on Pacific islands following human colonization [3–10] suggesting that biodiversity on many Pacific Islands is already substantially impoverished and/or that extant populations of species may currently be significantly reduced from their original size prior to human settlement.

**Fig 1:**
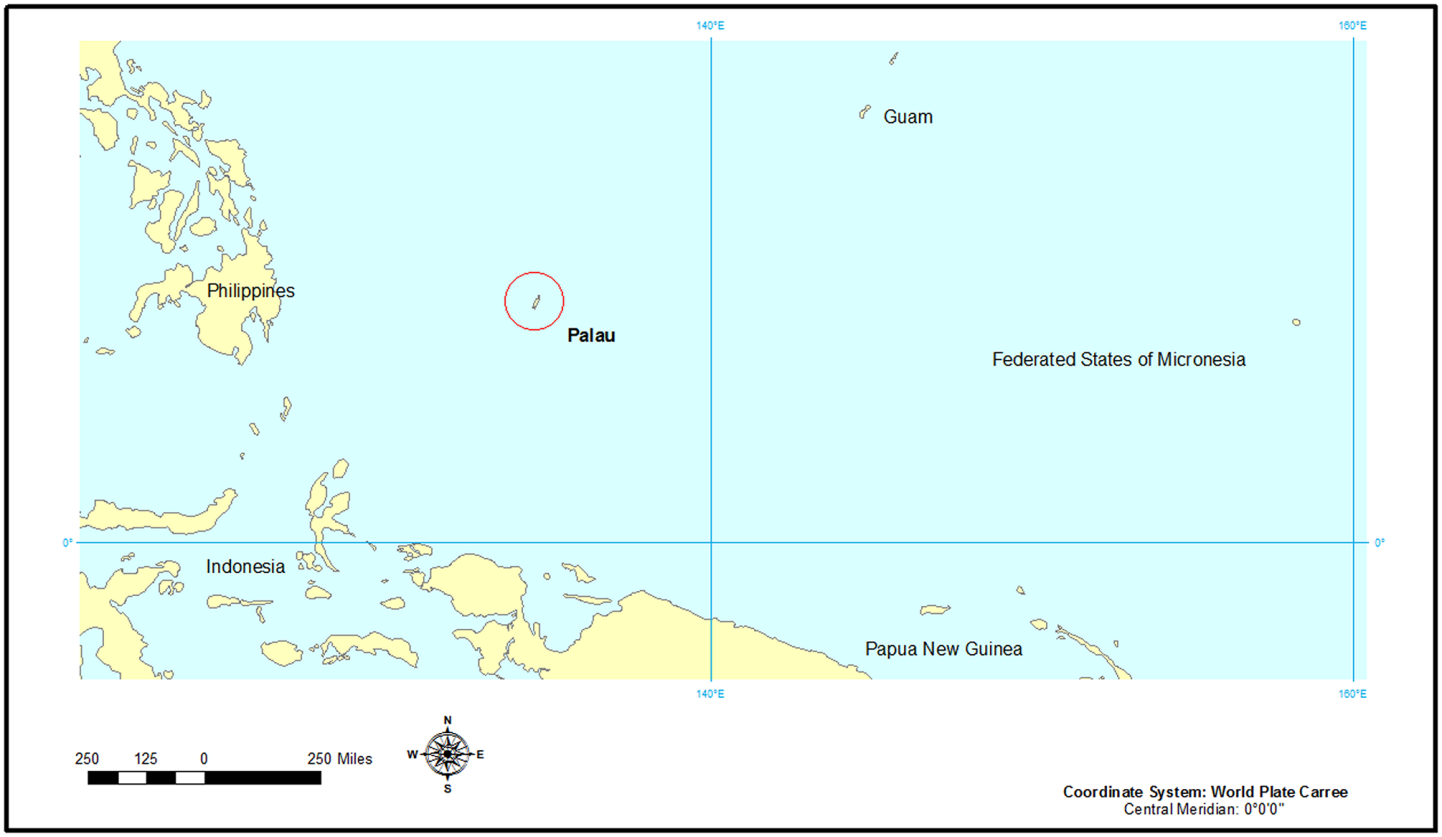
The archipelago of Palau, only 465 km^2^, occurs west of the Federated States of Micronesia (FSM), and southwest of the Guam and the Marianna Islands. Map created with ArcGIS, ArcMAP software.

The archipelago of Palau, Micronesia, in the western Pacific region (Fig 1) of the tropics is one such region. A recent study [11] demonstrated that the original forest habitat on the largest of Palau’s islands has declined by a minimum of 39% since the settlement of humans on the islands. Given the extinction trends for the Pacific islands it may be expected that some of Palau’s endemic plant species are already in an advanced state of decline. Due to the remote location of these islands, until very recently no detailed studies of the endemic plant species nor efforts to assess their threatened status had been undertaken. A preliminary assessment [12] concluded that although at least 61% of the endemic flora was considered Data Deficient using IUCN categories, one particular species, *Timonius salsedoi* Fosberg & Sachet, should be considered Critically Endangered due to its exceptionally small extant range.

Here we assess a species complex of *Timonius* DC. (Rubiaceae) in Palau with a specific focus on the taxonomic and conservation status of an allegedly Critically Endangered species [12], *Timonius salsedoi* Fosberg & Sachet. Prior to this study *T*. *salsedoi* was only known from the type collection and its inferred distribution was so rare and restricted that it qualified for Critically Endangered status on the IUCN Red List [13]. Assessing the conservation status of this species necessitated investigating its taxonomic status in relation to four other closely related *Timonius* species in the Palau archipelago. We conducted an intensive field survey of the potential range of *T*. *salsedoi*, a morphological review of new and existing collections and a molecular analysis of the Palau *Timonius* species using seven plastid loci including four universal plant DNA barcode loci [14].

This study is the first molecular assessment of species relationships within the genus *Timonius*. *Timonius* is the largest genus in the Rubiaceae tribe Guettardeae. *Timonius* comprises approximately 200 species of dioecious trees and shrubs [15] and has a distribution extending from the Seychelles eastward to Sri Lanka, Southeast Asia, Taiwan, Australia, and the Pacific. Although several subtribes of *Timonius* have been taxonomically revised, virtually no molecular phylogenetic work has been done to date within the genus [16]. All published sequences of *Timonius* found on GenBank are linked to studies that investigate intergeneric relationships within the family Rubiaceae, other Rubiaceae genera, or other ecological questions [17–23]. DNA barcodes in another Rubiaceae genus, *Hedyotis* L., were assessed and found to be 96–100% accurate at delimiting species [24] but since it is well known that plastid loci evolve at different rates in different lineages of vascular plants even within the same family [25], the same level of accuracy cannot be assumed for *Timonius*. Thus we tested the accuracy of seven loci to distinguish species in this genus.

### The Palau Timonius species complex

**Fig 2:**
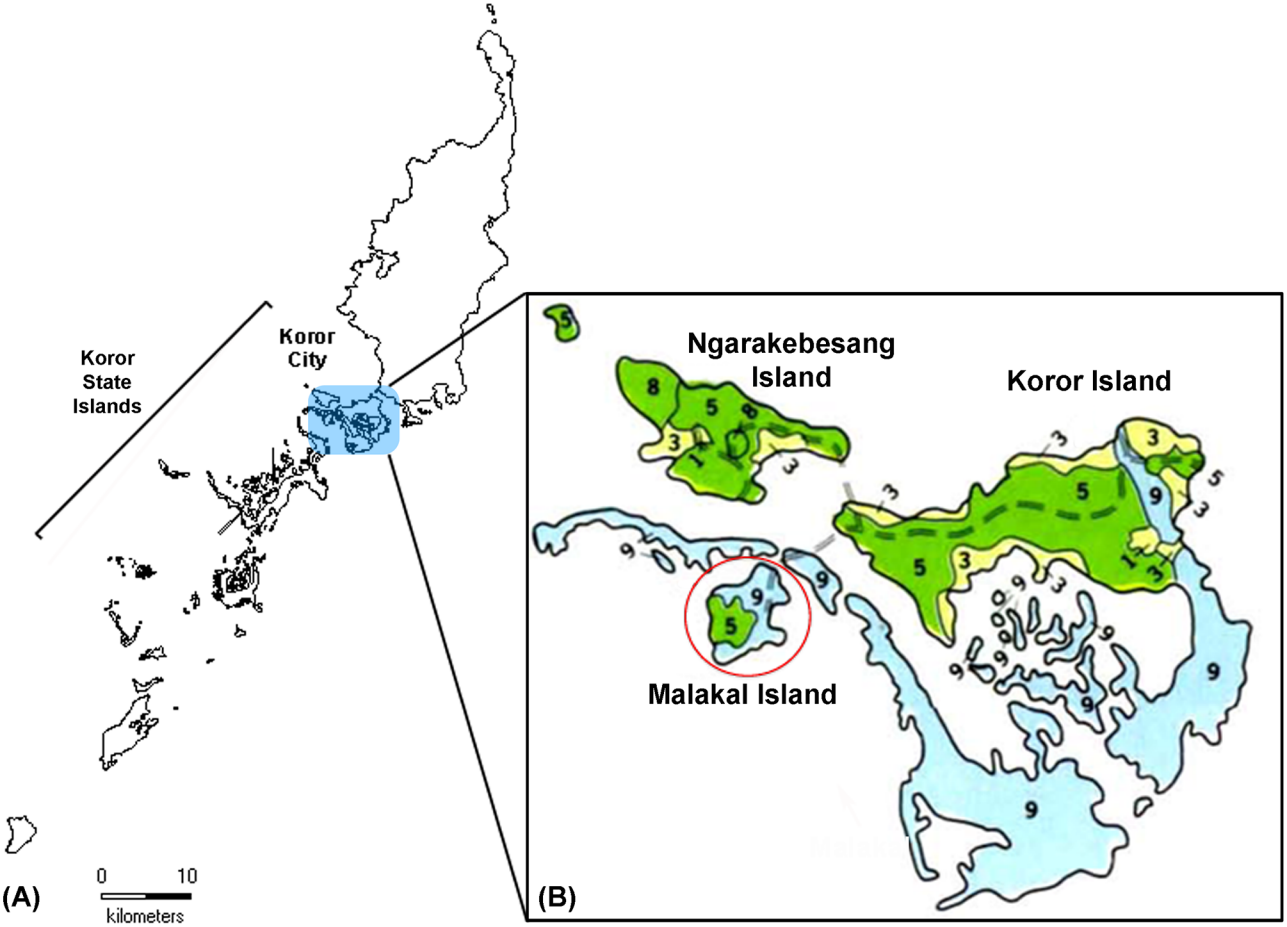
The Palau archipelago (A) is shown with the Koror State region in brackets and the Koror urban district highlighted in blue. Zoomed in view of the Koror urban district (B) is shown will soil types color-coded (map derived from USDA Soil Survey of Islands of Republic of Palau [28]). Limestone soils are shown in light blue, volcanic soils in green, and coastal and intertidal areas in yellow (numbers 1–9 indicate subcategories of these three soil types). Malakal Island is circled in red and the other two inhabited islands, Ngarakebesand Island and Koror Island are labeled.

Five species are included in the Palau *Timonius* complex. Of these species three taxa, *Timonius subauritus* Valeton, *Timonius mollis* Valeton, and *Timonius corymbosus* Valeton var. *takamatsui* Fosberg and Sachet are relatively well known, are easy to distinguish, and are represented by many collections. Two species, *Timonius salsedoi* and *Timonius korrensis* Kaneh., are poorly known, being represented by few collections in herbaria and having poorly understood taxonomic boundaries. All are considered endemic to Palau, and three species, *T*. *subauritus*, *T*. *mollis*, and *T*. *corymbosus* have further endemic varieties or subspecies also endemic to Palau. *Timonius salsedoi* was described as taxonomically close to both *Timonius subauritus* Valeton and *Timonius mollis* Valeton [26,27]. Until very recently *T*. *salsedoi* was known only from the type collection from Malakal Island in Palau. This island is only 0.85 km^2^ in size and partly urbanized with only a small section of extant remaining forest habitat (See Fig 2, forested area shaded green). The progressive decline in suitable habitat in the area and the species’ exceptionally small population size qualify it under IUCN Red List Criteria B1ab(iii) and B2ab(iii) for Critically Endangered Status [12].

Collections and available information on both *T*. *salsedoi* and *T*. *korrensis* is minimal. In addition, the extensive urbanization in the Koror municipality over the past century may have reduced the distribution of these taxa, further complicating a clear understanding of their delimitation. The other three species have well documented distributions and are easy to distinguish from each other. *Timonius corymbosus* Valeton has two recognized varieties. One is commonly found throughout Palau’s limestone islands, the other is only known from the lectotype collected from Koror. It tends to be more abundant along the inland karst slopes and ridges but can also occur on the exposed coastal cliffs. *Timonius subauritus* Valeton and *Timonius mollis* Valeton are both common throughout Palau’s volcanic islands. They both occur as understory trees in well-developed forest as well as in savanna. The former has two recognized varieties, *Timonius subauritus* var. *strigosus* Fosberg and Sachet and *Timonius subauritus* var. *subauritus* Fosberg and Sachet and the latter has three, *Timonius mollis* var. *submollis* Fosberg & Sachet, *Timonius mollis* var. *villosissimus* (Kanehira) Fosberg and Sachet, and *Timonius mollis* var. *mollis* Fosberg & Sachet.

The varieties of *T*. *mollis* and *T*. *subauritus* proposed by Fosberg and Sachet [27] are separated only by the size of leaves and flowers and density of pubescence. Fosberg and Sachet [26] state that no information exists whether or not these varieties occur in different ecological situations, but recent field observations outlined below suggest that they may indeed be ecotypes. Considering Fosberg and Sachet’s *T*. *salsedoi* was only described from one fertile female specimen, and it is described as a small shrub within a species complex of trees, it raises the question whether this specimen may fall within the known variation of either *T*. *mollis* or *T*. *subauritus*. This question was deemed worthy of testing due to the conservation implications required if *T*. *salsedoi* is a distinct species.

Here careful study of morphology both in the field and herbarium, as well as genetic analysis of seven plastid loci are used to distinguish the five species of *Timonius* in Palau. The primary motive of this study was to determine if the DNA barcode and additional plastid loci confirm that *Timonius salsedoi* is distinct from *T*. *subauritus* and/or *T*. *mollis*, as suggested by morphology. In the process of investigating this primary question, however, morphological evidence from historic and recent collections was uncovered that suggested the necessity of revising additional species within the Palau complex.

## Materials and Methods

### Field survey and sampling

No animals or humans were used during this study. Field permits and access to study sites in Palau were granted by Koror State and Airai State in collaboration with the Belau National Museum.

A comprehensive survey of suitable habitat for *Timonius* on Malakal island, the only know locality of *T*. *salsedoi*, was conducted. A total of 15 individuals of *Timonius* identified as *Timonius* sp. were sampled from Malakal for molecular and morphological study. Two of these samples were notably distinct from the other 13 in having three-branched female inflorescences with dense whitish pubescence on the fruits. Ten individuals of *Timonius mollis* and 15 of *Timonius subauritus*, were sampled from Babeldaob island, which is separated from Malakal by the capital city of Palau, Koror, and a large channel within the Palau lagoon. These individuals were selected to represent the diversity of varieties/subspecies described; plants with pubescent and glabrous foliage, different sized flowers, and from different ecological conditions; in savanna with open canopy and in understory of closed forest. Three individuals of *Timonius corymbosus* var. *takamatsui* from the limestone Rock Islands were also sampled. A total of 53 new *Timonius* collections were obtained.

To test the robustness of Fosberg and Sachet’s varieties of *T*. *subauritus* and *T*. *mollis*, three to six representative specimens were obtained from each of several different ecological conditions: plants in full sun, full shade, cuttings sampled from trees from sun exposed canopy, and multiple samples from trees with branches in both the shaded understory and sun exposed canopy. These were than assessed against the characters used to delimit the varieties of the two species [26].

### Morphological study

In addition to the 53 individuals newly sampled from the wild, collections of *Timonius* from the Palau Islands from the Bishop Museum Herbarium, Hawaii (BISH), the US National Herbarium (US), the Belau National Museum Herbarium, the University of Tokyo’s two separate Herbaria in Japan (TI, TOFO) and the Herbarium of the Kyushu University Museum (FU) were examined. Importantly, these included Kanehira specimens held at the University of Tokyo and Kyushu University that were not studied by Fosberg and Sachet for their revision of the genus for the Flora of Micronesia [26,27].

### Molecular study

A preliminary subset of 19 of the tissue samples was selected for DNA extraction including seven samples of *Timonius* sp. from Malakal island, four samples of *Timonius mollis*, six samples of *Timonius subauritus*, and two samples of *Timonius corymbosus* var. *takamatsui*.

Total genomic DNA was extracted using the Qiagen DNeasy 96 plant kit. PCR was performed for four commonly used plant DNA barcode loci (*rbcLa*, *matK*, *trnH-psbA*, *ITS*) plus three additional plastid markers (*trnL-trnF* intergenic spacer, *atpB-rbcL* intergenic spacer, and *petD*). A complete list of the primers used with their associated references is provided in Table 1. A list of the PCR mixtures and thermal profiles for each locus is also provided (S2 Table).

**Table 1:** List of primers used for each locus with references.

Locus	Forward Primer	Reverse Primer	Reference
*trnH—psbA*	trnHf_05	psbA3 f	[29,30]
*matK*	3F_KIM_f	1R_KIM_r	unpublished
*ITS*	ITSP17	ITS26S82R	[31]
*rbcLa*	rbcLa_f	rbcLa_r	[32,33]
*trnL—trnF*	trnLF-c	trnLF-f	[34]
*atpB—rbcL*	377 (F)	2607 (R)	[35]
*petD*	PIpetB1365F	PIpetD738R	[36]

Sanger sequencing was performed by the Australian Genome Research Facility (AGRF). Trace files were assembled and edited in DNA baser v.3.5.3 (http://www.dnabaser.com/) and checked manually for ambiguous bases. Sequences were aligned using MAFFT online v.7 (http://mafft.cbrc.jp/alignment/software/)then manually inspected for informative sites in BioEdit v.7.1.3 (http://www.mbio.ncsu.edu/bioedit/bioedit.html). In total 75 new nucleotide sequences were generated and submitted to GenBank (S3 Table).

Individual locus alignments were then concatenated into one alignment and analyzed in MEGA v.5 [37]. To assess total genetic distance and relationships between the taxa, maximum likelihood, neighbor-joining, and maximum parsimony trees were constructed from the seven-locus alignment with bootstrap tests of 1,000 replicates each. An additional neighbor joining analysis was conducted using only the four DNA barcode loci. The evolutionary history for the maximum likelihood tree was based on the Tamura-Nei model with branch lengths measured in the number of substitutions per site. Initial trees for a heuristic search were obtained automatically by applying neighbor joining algorithm to a matrix of pairwise distances estimated with the maximum composite likelihood approach then identifying the topology with superior log likelihood value. Evolutionary distances for the neighbor joining tree were computed using the number of differences method [38] which counts the number of base differences per sequence. The maximum parsimony tree was obtained using the Close-Neighbor-Interchange algorithm [38] with search level one in which the initial trees were obtained with the random addition of sequences (10 replicates).

## Results

### Morphological data

A summary of important morphological distinctions for each species examined is presented in Table 2. Fruit characters, pubescence of leaves at maturity, and substrate upon which the plants grow were found to be the most reliable characters for differentiating species. Leaf shape and size is widely variable within species and staminate inflorescences are hard to distinguish clearly between species. A more complete discussion including a list of the herbarium specimens and the specific morphological characters examined is provided as Supplementary Data (S1 Table).

**Table 2:** Summary of important morphological characters that distinguish the Palau *Timonius* species.

	*T*. *corymbosus*	*T*. *korrensis*	*T*. *mollis*	*T*. *salsedoi*	*T*. *subauritus*
No. fruits per inflorescence	unknown	3	1	3	1
Fruit pubescence	unknown	glabrous	glabrous-dense	short, whitish	glabrous
Fruit shape	unknown	mammilate	globular	globular	globular
Persistent Calyx	unknown	short, erect	long, recurved	short, erect	short, erect
Leaves at maturity	densely pubescent	glabrous	glabrous-densely pubescent	glabrous, whitish pubescentunderside midrib	glabrous, partially pubescent on underside midrib
Substrate	unknown	limestone	volcanic	volcanic	volcanic

### Molecular data

A total of 15 of the 19 samples were successfully amplified for all loci. Out of the four DNA barcode loci, a total of four informative base pairs were identified (Table 3). The *matK* locus had no variable sites whereas *rbcLa*, which is usually less variable, had one. ITS had one informative base pair and *trnH-psbA* had two including one long indel of 23 base pairs which was coded as one evolutionary event. Coding for this indel was tested by assessing all possible base pair changes and constructing a separate neighbor-joining tree for each possible base pair change. The results were unaffected by how the indel was coded; however, coding it provided additional support for distinguishing one of the taxa from all other species.

**Table 3:** Summary of informative characters across all species for each locus sequenced and the number of species they distinguished.

Locus	Variablesites	Gaps/Indels	Informativecharacters	Species distinguished
*rbcL*	1	0	1	2
*matK*	0	0	0	0
*trnH-psbA*	1	1	2	3
*ITS*	1	0	1	2
*trnL-trnF*	0	0	0	0
*atpB-rbcL*	2	0	2	2
*petD*	0	0	0	0
4 locus barcode	3	1	4	4
All 7 loci concatenated	5	1	6	4

**Fig 3:**
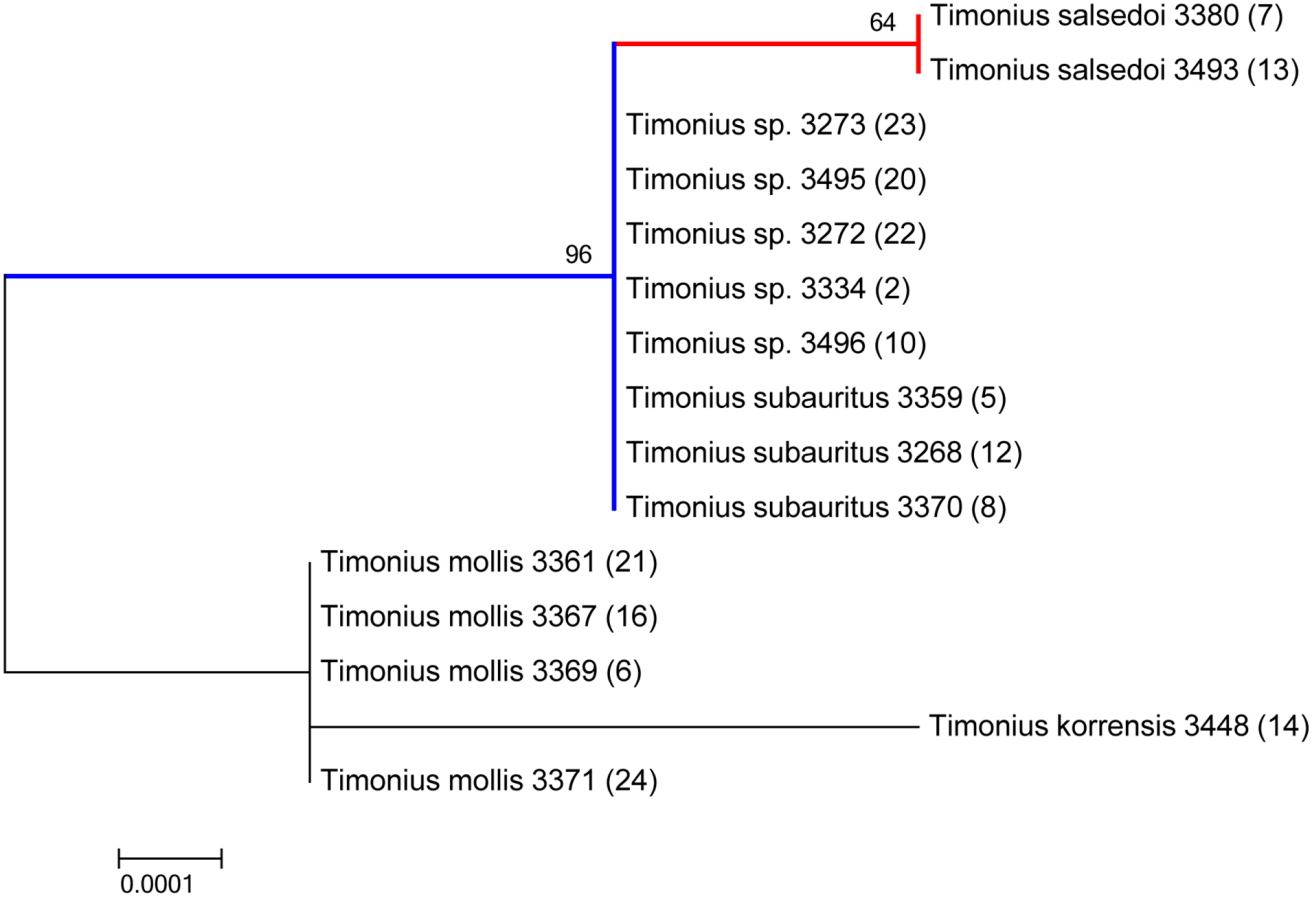
Maximum likelihood tree from seven-locus DNA barcode alignment (*rbcL*, *matK*, *ITS*, *trnH-psbA*, *trnL-trnF*, *petD*, *atpB-rbcL*) with bootstrap support values at branch nodes and species followed by collection number (DNA sample number) at the branch tips. The tree with the highest likelihood (-4639.8901) is shown and drawn to scale, with branch lengths proportional to the number of substitutions per site.

**Fig 4:**
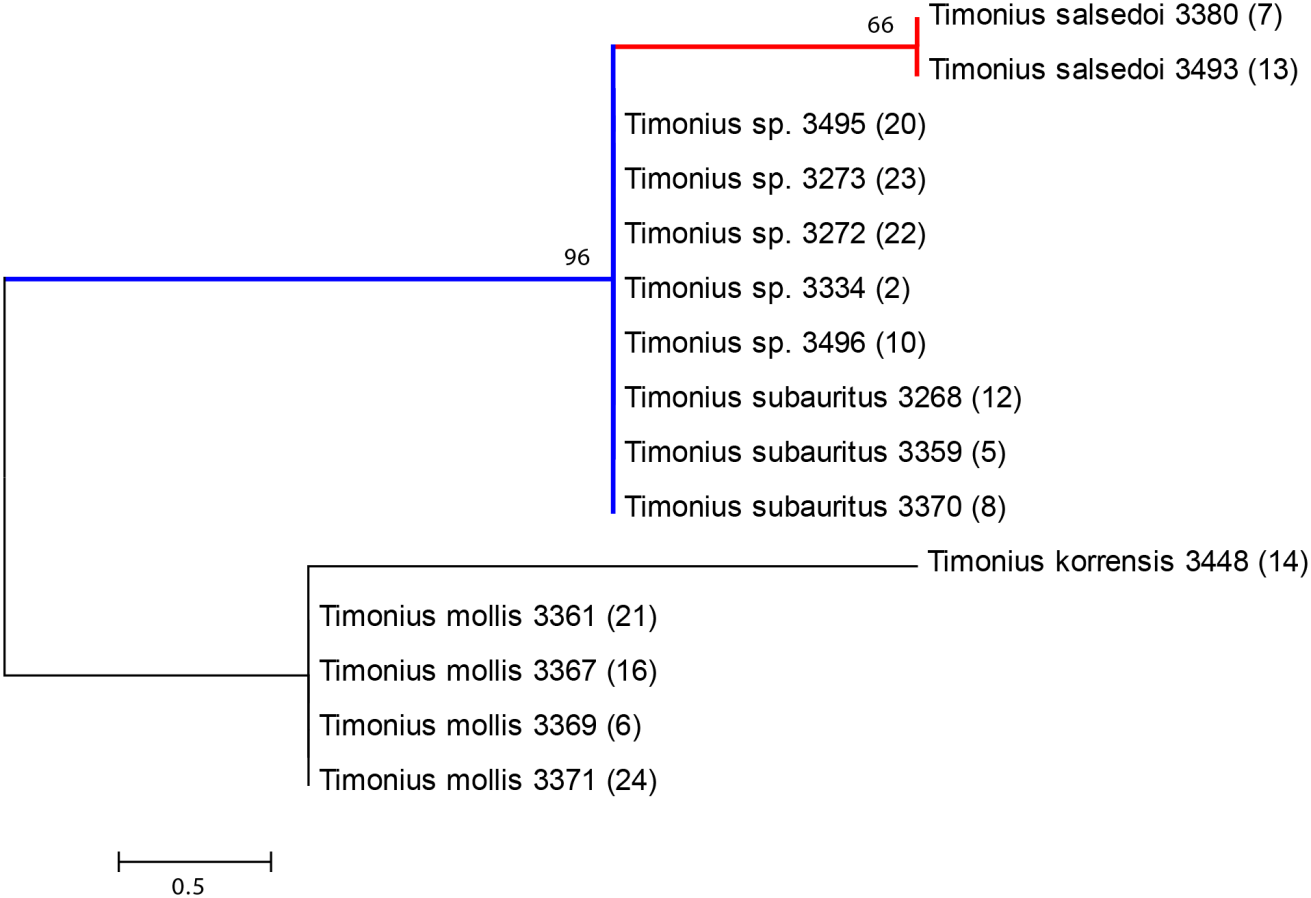
Neighbor-joining tree from seven-locus alignment (*rbcL*, *matK*, *ITS*, *trnH-psbA*, *trnL-trnF*, *petD*, *atpB-rbcL*) with bootstrap support values at branch nodes and species followed by collection number (DNA sample number) at the branch tips. The tree is drawn to scale, with branch lengths in the same units as those of the evolutionary distances used to infer the phylogenetic tree.

**Fig 5:**
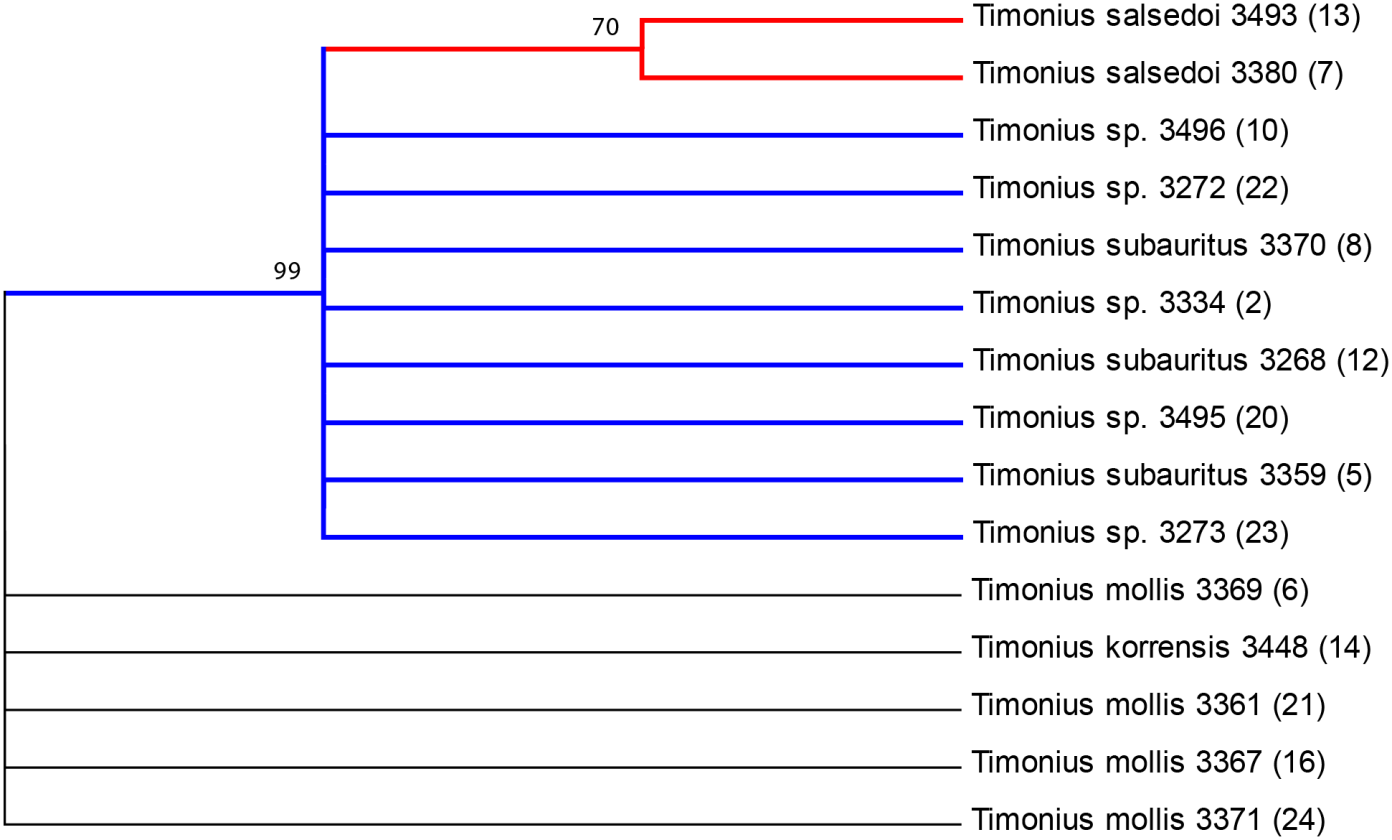
Maximum parsimony tree from seven-locus alignment (*rbcL*, *matK*, *ITS*, *trnH-psbA*, *trnL-trnF*, *petD*, *atpB-rbcL*) with bootstrap support values at branch nodes and species followed by collection number (DNA sample number) at the branch tips. The tree shown is the bootstrap consensus tree inferred from 1,000 replicates, Branches with <50% support are collapsed. The lineage of *T*. *subauritus* is highlighted in blue and *T*. *salsedoi* is highlighted in red.

None of the barcode loci performed particularly well alone. In particular the official DNA barcode regions *rbcL* and *matK* performed poorly together as a two-locus barcode with only one informative site distinguishing two out of five alleged species. Out of the three additional loci tested, only the *atpB-rbcL* intergenic spacer showed any variation. This locus contained two informative sites, which provided additional support for the relationships identified by the four-locus barcode. All possible three-locus combinations using *rbcL* and *matK* plus any third locus identified three species maximum whereas the four-locus combination including *rbcL*, *matK*, *trnH-psbA*, and *ITS* accurately identified four species but using all seven loci combined increased support for the tree topology (Figs 3, 4 and 5).

All three phylogenetic methods distinguish *T*. *salsedoi* from *T*. *subauritus* and strongly support a clade that includes these two species as divergent from the other Palau species. The maximum parsimony tree (Fig 5) shows 99% support for a clade including *T*. *subauritus* and *T*. *salsedoi* while both the maximum likelihood and neighbor joining trees show 96% support. *T*. *salsedoi* is distinguished from *T*. *subauritus* with 70% support in the maximum parsimony tree, 64% support in the maximum likelihood tree, and 66% in the neighbor joining tree. Poor support is found for distinctions between *T*. *korrensis* and *T*. *mollis* even though these two taxa are very different morphologically.

### Taxonomy

On the basis of our field inventory and combined morphological and molecular analysis we advocate the recognition of five species among Palau *Timonius*. These are as follows.

**Timonius corymbosus** Valeton, Bot. Jahrb. Syst. 63(4): 308. 1930

Type: Koror, Ledermann 14051 (lectotype: B!)

This species is only known from the type specimen collected in 1914, which is male. All herbarium specimens identified as *T*. *corymbosus* var. *corymbosus* subsequently have been mis-identified. This species is morphologically similar to *T*. *korrensis* but notably distinct in having densely pubescent leaves and inflorescences. The fruits and female fertile parts of this species remain unknown. Its distribution and substrate preference also remain unknown due to the limited information recorded on the type.

**Timonius korrensis** Kanehira, Bot. Mag. (Tokyo) 1931, xlv. 351

Type: Koror State, Kanehira 105 (syntype: FU!); Kanehira 460 (Syntype: FU!, TOFO!)

*Timonius corymbosus* var. *takamatsui* Fosberg and Sachet, Micronesica 20:159, 1987.

Type: Koror state, Takamatsu 1478 (holotype: BISH!)

This species is distinguished by its three-branched, glabrous, female cymes, its irregularly mammillate fruits, and its distribution being restricted to limestone substrates. Review of new specimens not available to Fosberg and Sachet necessitates incorporating their former name *T*. *corymbosus* var. *takamatsui* into *T*. *korrensis*.

**Timonius mollis** Valeton, Bot. Jahrb. Syst. 63(4): 308. 1930

Type: Melekeok, Babeldaob, Kanehira 5051 (neotype: FU)

*Timonius villosissimus* Kaneh., Bot. Mag. (Tokyo) 1934, xlviii. 923.

Type: Aimeliik, Kanehira 2305 (lectotype: US!, isolectotypes: FU!, NY!)

*Timonius mollis* var. *submollis* Fosberg & Sachet, Micronesica 20:161, 1987.

Type: Ngarakabesang, Fosberg 25630 (holotype: US)

*Timonius mollis* var. *villosissimus* (Kanehira) Fosberg and Sachet, Micronesica 20:161, 1987.

Type: Aimeliik, *Kanehira 2305* (FU holotype!, US, isotype!)

This species is distinguished by its solitary fruits borne in the leaf axils, its persistent pubescence on the leaves, and its characteristic long, recurved calyx persistent on mature fruits. *T*. *mollis* is closest to *T*. *subauritus* and can be difficult to tell apart for infertile plants due to the intermediate stages of pubescence occurring on plants of *T*. *subauritus*. The long recurved persistent calyx however easily distinguishes it. The boundaries separating the three varieties of *T*. *mollis* are inconsistent across available specimens thus we propose a more variable, *sensu lato*, concept of *T*. *mollis*.

**Timonius salsedoi** Fosberg and Sachet, Micronesica 20(1–2): 162. 1987

Type: Malakal, Fosberg 47508 (holotype: US!; isotype: BISH!)

This species is distinguished by its three-fruited cymes borne on long peduncles, fruits with dense whitish pubescence, and its short erect persistent calyces. It is known from only three collections from the volcanic island of Malakal in the Koror urban district.

**Timonius subauritus** Valeton, Bot. Jahrb. Syst. 63(4): 307. 1930

Type: Koror, Ledermann 14046 (lectotype: B!); Ledermann 14196 (paratype: B!)

*Timonius subauritus* var. *strigosus* Fosberg and Sachet, Micronesica 20:163, 1987.

Type: Southern Babeldaob, Hosokawa 7279 (holotype: A, isotype: US)

This species is distinct by its solitary glabrous fruits borne in the leaf axils, and its short erect calyxes persistent on the fruits. Its leaves can be pubescent at juvenile stages and in the shade or forest understory. Pubescence can persist on the underside mid-rib of leaves but plants exposed to full sun tend to be glabrous. Previously recognized varieties based on this variable pubescence were found to co-occur on same plants in shaded and sun-exposed branches and are thus synonymized here.

## Discussion

The results indicate that the two new collections identified as *T*. *salsedoi* from Malakal Island are distinct genetically and morphologically from the other three species sampled. It also, most importantly, confirms that all other individuals of *Timonius* sp. found on the island of Malakal are *T*. *subauritus*. The closest relative to *T*. *salsedoi* is *T*. *subauritus* as expected and the two apparently co-inhabit the island of Malakal. Five of the other undetermined samples from the Malakal Island formed a clade with *T*. *subauritus* and were identical across all seven loci to *T*. *subauritus* (Figs 3, 4 and 5). To be certain of this uniformity across the remaining *Timonius* samples of the Malakal population the remaining eight samples from Malakal and additional samples of *Timonius subauritus* and *Timonius mollis* were sequenced for the *atpB-rbcL* intergenic spacer, the region with the highest number of variable base pairs. All of these samples from Malakal were identical to *T*. *subauritus*, distinguishing them from *T*. *mollis*, *T*. *korrensis* and *T*. *salsedoi*. Since the island was intensively surveyed for *Timonius*, this suggests that there may be only two remaining trees of *T*. *salsedoi*.

In the phylogenetic analysis the high bootstrap support for the clade including *T*. *subauritus* and *T*. *salsedoi* separates a second clade including *T*. *mollis* and *T*. *korrensis* (Figs 3, 4 and 5). This result separates *T*. *salsedoi* from *T*. *korrensis* despite their similarity in having three-flowered inflorescences. The inferred evolutionary relationships however do not incorporate morphological data and are likely to be improved with increased taxon sampling across the Pacific and from potential dispersal source areas such as New Guinea, Philippines, and Indonesia. Despite this, the results support our study of the morphology and conclusions on species boundaries within the archipelago. DNA barcodes identified four species from our samples, each distinguished by more than one base pair. *T*. *korrensis* is distinguished from *T*. *mollis* by two base pairs and from *T*. *subauritus* and *T*. *salsedoi* by five base pairs. *T*. *mollis* is distinguished from *T*. *subauritus* and *T*. *salsedoi* by three base pairs. *T*. *subauritus* and *T*. *salsedoi* are the closest genetically with only one base pair distinguishing them although they are quite different morphologically.

Our study of the morphology can clearly distinguish the same taxonomic units identified by the molecular data. *T*. *mollis* and *T*. *subauritus* are very close, morphologically. Both have single fruits at maturity, have similar foliage, and they occur in the same habitats. They are distinguished by their mature fruits, persistent calyces, and by the pubescence on mature leaves. *T*. *salsedoi* and *T*. *korrensis* are somewhat similar in morphology, having three branched inflorescences. They are strongly distinguished by the size of leaves, length of inflorescence stalks, and the shape and pubescence of fruits.

The validity of infraspecific taxa in *T*. *subauritus* and *T*. *mollis* is also called into question here by recent field observations on the variability of pubescence between plants in different ecological situations and the lack of genetic variation found between samples. Further work is required to determine whether this variation can be attributed to ecological factors or hybridization. Until then we propose that infraspecific taxa are not recognized within *T*. *subauritus* and *T*. *mollis*.

Our primary question was to assess whether *T*. *salsedoi* should be treated as a distinct species or whether it fits within the observed plasticity of *T*. *subauritus*. Since our study included samples representing the different varieties of *T*. *subauritus* and found no variation between them but distinguished *T*. *salsedoi*, Fosberg’s concept of *T*. *salsedoi* is supported. We also obtained new morphological support for *T*. *salsedoi* with the two new collections. The type of *T*. *salsedoi* has solitary fruits only. Our new collections, however, show that *T*. *salsedoi* can have flowers and fruits both solitary and borne in threes on the same plant suggesting that solitary flowers may be an ancestral character in the Palau *Timonius* complex.

With the available data we are unable to completely infer the evolutionary relationships of the Palau species, therefore the direction of character evolution and the number and origin of lineages on Palau is largely unknown. Broader taxon sampling including from New Guinea, the Philippines, and other Pacific Islands, and the use of more variable DNA markers will enable such questions to be addressed more thoroughly.

Review of herbarium material not available to the original authors of *T*. *corymbosus* var. *takamatsui* strongly supports inclusion of the name into *T*. *korrensis*. This species is highly distinctive morphologically and appears to be geographically restricted to the limestone islands.

This small case study of closely related species on a Pacific archipelago is the first exploration of genetic variation within the genus *Timonius*. Recommended markers for additional work in *Timonius* include *rbcL*, *trnH-psbA*, *ITS*, and *atpB-rbcL*. Although the four routine DNA barcode loci were effective alone, and may be useful for studies where DNA barcode data are available across a bioregion for a suite of species, we found *atpB-rbcL* to be a useful additional marker for more specialized work in *Timonius*. The loci *matK*, *trnL-trnF*, and *petD* did not provide informative characters for the taxa in this study.

### Conservation Implications

This study indicates that *Timonius salsedoi* is genetically distinct, and may be extinct or nearly so. Only two trees were located in the initial survey in early 2012. In 2014 the island was revisited and these two trees had been replaced by a large tree-fall break in the canopy likely caused by typhoon Bopha in December 2012. No evidence of regrowth or emergent seedlings of *T*. *salsedoi* was found in the same location. A more intensive follow up inventory is urgently required to locate any seedlings and additional mature individuals that may have been missed in the initial inventory. Given the genetic evidence presented here for its distinction as a species, its geographically restricted range, and the results of the survey indicating there were only two individual trees remaining in 2012 (these are now dead) *Timonius salsedoi* qualifies as a Critically Endangered Species under three separate IUCN criteria: B1&2ab(v), C2a(i), and D. Both the extent of occurrence and area of occupancy is less than 10 km^2^, the population exists only at one location, and has been observed to be declining in the number of mature individuals (Criterion B1&2ab(v)). The total population of mature individuals is also certainly less than 50 (Criteria C2a(i) and D).

Recent studies suggest that loss of species here has global significance. Palau and the Micronesia region as a whole are renowned for their high levels of species endemism having the highest count of endemic plant species per square kilometer of all globally recognized island biodiversity hot spots [8]. Palau has by far the highest number of endemic plants within Micronesia accounting for 37% of the total for the bioregion. Although species richness of the islands is comparatively poor to other tropical continental islands and regions their importance has not been overlooked. A recent study on global patterns of forest bird diversity [39] ranked Palau’s forests a number two globally in an impact score for conservation status of the world’s forest birds even though bird species richness is relatively low. The narrow distribution of these birds gave them high enough value to rank number two compared to other regions with much higher species richness. These data on forest bird diversity predict that protecting a very small area of forest in Palau will return high value for biodiversity conservation and that loss of a small amount of forest here may result in loss of important species not represented anywhere else in the world. The outcomes of the current study through observation and a traditional taxonomic investigation supports the theoretical basis outlined by the forest bird study. The loss of forest on the islands of Koror and Malakal is seemingly insignificant in its size but was enough to tip the balance for an entire species, *Timonius salsedoi*.

Ironically, prior to embarking on this project, in an attempt to hasten progress on assessing the conservation status of tropical plant species, the first author wrote in a separate article that “it is probable that the loss of many species of Oceania’s flora and fauna have gone unrecognized and unrecorded” [11]. We set out in this project to verify the conservation status of a species that was allegedly very rare and at risk due to its proximity to an urban area. In the process, we witnessed first-hand the disappearance of the only two known individuals represented by *Timonius salsedoi*. This study emphasizes the fragility of tropical island species and how important it is to continue research on their basic biology and taxonomy. The former range of *Timonius salsedoi* cannot be known but it is almost certain that it was once more widely distributed across the island of Malakal and very likely also the island of Koror, which is now entirely urbanized. Unfortunately for *Timonius salsedoi*, and the inhabitants of Palau, knowledge of this unique species’ existence has come late. It is hoped that this study will serve as a reminder of how little we know about the Earth’s tropical plant species and the significant role taxonomy plays, utilizing both traditional morphological and modern molecular and DNA barcoding methods, in their conservation.

## Supporting Information

S1 Table Supporting information. Specimen data and field observations.
(DOCX)

S2 Table PCR formulas. List of PCR mixtures and thermal profiles used for each locus.
(DOCX)

S3 Table DNA sequence list. List of GenBank accession numbers for all nucleotide sequences generated and used in the analysis.
(XLSX)
