# Supplementary material for: DNA Barcodes Confirm the Taxonomic and Conservation Status of a Species of Tree on the Brink of Extinction in the Pacific
Source: PLoS One. 2016 Jun 15;11(6):e0155118. doi: 10.1371/journal.pone.0155118 (PMC4909193; doi:10.1371/journal.pone.0155118)
Supplement: S1 Table — Specimen data and field observations. (DOCX) [file pone.0155118.s001.docx]

**Specimen data and field observations**

**Timonius corymbosus** Valeton

Many specimens of *T. korrensis* are available for study and have been observed in the field throughout its range. To date no pubescent specimens of *T. korrensis* have been observed. The fruits of *T. corymbosus* however, which contain the most reliable diagnostic characters in this species complex, are unknown. An additional challenge to understanding this species is the poor locality data recorded on the only known specimen. Ledermann only noted that the collection was made in Koror. Koror is a city, and island, and a state encompassing hundreds of islands of both limestone and volcanic origin (Figure 2). If the specimen was collected from Koror Island, the species is likely to be extinct since there is very little forest remaining on the island. Conversely if the specimen was collected somewhere else in the Koror municipality, which includes the Rock Islands, then there are likely more to be found. Until further material becomes available matching *T. corymbosus*, particularly in the fruiting stage, the name must be retained.

SPECIMENS EXAMINED: PALAU: Koror: *Ledermann 14051*

**Timonius korrensis** Kanehira

Specimens observed at the University of Tokyo and the Kyushu University Museum in Fukuoka, indicated the need for review of the taxonomy of this species. Of particular importance was the syntype (*Kanehira 460*) and additional Kanehira collections not seen by Fosberg and Sachet (1987, 1993)*.* Fosberg and Sachet differentiated their species *T. corymbosus* var. *takamatsui* from *T. korrensis* by having more slender and more times ramified inflorescences, smaller flowers, and more slender and more branched pistillate cymes. A recent extensive survey of Palau’s limestone islands [32] however, indicated that slenderness and number of inflorescence ramifications varied considerably even within cuttings taken from the same tree (*Costion 3513, 3448*). The morphology of fruits present on all Kanehira collections is also unmistakable matching identically to Fosberg’s description of the fruits of *T. corymbosus* var. *takamatsui*. The “irregularly mammillate” fruits are a diagnostic character of this species distinguishing it from all the other Palau taxa. Most importantly Fosberg and Sachet [21] described *T. korrensis* as having once-dichotomous pistillate cymes that are shorter than *T. corymbosus* var. *takamatsui.* This is consistent with the material that was available to them however two Kanehira collections, *2444* and *2466* clearly show *T. korrensis* with a twice-dichotomous pistillate cyme. Since Kanehira’s description of *T. korrensis* precludes and is based on material identical to Fosberg and Sachet’s *T. corymbosus* var. *takamatsui* the latter name must be re-incorporated into the former.

SPECIMENS EXAMINED: PALAU: Koror State: Shearard & Spence 75 (US); Rock Islands: Kanehira 105 (FU, syntype); Kanehira 460 (FU, TOFO, syntype); Kanehira 2436, 2466 (FU, TOFO); Kanehira 2462 (FU, US); Kanehira 2444, 4660 (FU); Raulerson 16994 (US); Rinehart LR 16812 (US); Takamatsu 1458 (US); Takamatsu 1452 (BISH); Costion 3513 (CNS, BNM); Costion 3448 (CNS, BNM); Hobdy 1445 (BISH); Fosberg 47569 (BISH); Peleliu:; Eastlick LR 22521 (US); Eastlick LR 22552 (US); Hosaka 3423 (US); Takamatsu 1786 (BISH); Angaur: Canfield 254 (US).

**Timonius mollis** Valeton

Three varieties of *T. mollis* were upheld by Fosberg and Sachet [20] separated primarily by the size of leaves and flowers with a large flowered, large leaved variety, a small flowered, small leaved variety, and one in between. The characters separating these varieties are very difficult to recognize in the field and do not hold up when cross comparing specimens across herbaria. Fosberg and Sachet doubted the characters used by Kanehira [33] for distinguishing *T. villosissimus* but deemed the dense pubescence worthy of varietal separation. There are specimens of *T. mollis* that are more densely pubescent than others but there are no clear lines to draw between the proposed varieties. Further more, no ecological information to date has been recorded to determine other possible causes of leaf size and pubescence such as indicated for its closest relative *T. subauritus* (see below). Since the boundaries separating the described varieties of *T. mollis* are nebulous and therefore of limited use, recognizing a more variable, *sensu lato*, concept of *T. mollis* may be preferred.

SPECIMENS EXAMINED: PALAU: Babeldaob: Flynn 6400, 6406 (US); Fosberg 32474, 32586, 47690 (US, BISH); Kanehira 415, 2055, 2056, 5051 (FU); Kanehira 2305 (FU, US); Hosokawa 9138 (US); Costion 3339, 3360, 3361, 3367, 3368, 3369, 3371, 3375, 3376, 3434 (CNS, BNM); Ngarakebesang: Costion 3498, 3499, 3500, 3502 (CNS, BNM).

**Timonius salsedoi** Fosberg and Sachet

Two of the 15 new collections of *Timonius* obtained during the survey of Malakal Island while searching for *T. salsedoi* (*Costion 3380, 3493*) are clearly distinct from all other collections from the island. Initially a match with *T. salsedoi* was disregarded due to its distinct 3-branched cymes on long peduncles not being consistent with Fosberg’s circumscription of the species having solitary pistillate flowers. The two new collections however include immature two-branched inflorescences fitting Fosberg’s description of *T. salsedoi* as well as more mature 3 branched cymes collected from the same tree. Review of the holotype (US) and isotype (BISH) of *T. salsedoi* indicated a good match, particularly with the distinct dense whitish pubescence present on the fruits, underside leaf mid-rib, and veins as well as the shape and size of the leaves. The whitish pubescence on the fruits distinguishes these three collections strongly from *T. subauritus,* which is known to have pubescence on the leaf underside veins and branches of new growth, but not on the fruits. *T. korrensis* has distinctly glabrous fruits. *T. mollis* does have pubescent fruits, however its distinctive, long, recurved, persistant calyx does not match the persistant calyx of *T. salsedoi* which is notably shorter and more erect. Furthermore, *T. mollis* is well collected and does not have 3-branched cymes. Although the fruits of *T. corymbosus* are not known, the leaf size and shape is very different. The new collections obtained give a stronger basis for the morphological distinction of *T. salsedoi.*

SPECIMENS EXAMINED: PALAU: Malakal: Fosberg 47508 (US, BISH); Costion 3380 (US, BNM); Costion 3493 (US, BNM)

**Timonius subauritus** Valeton

Currently this taxon has two recognized varieties that are distinguished based on pubescence. Pubescent specimens were observed in the field and collected on new growth of mature plants that are otherwise glabrous on mature branches. Plants with branches in the canopy more exposed to the sun were observed to have leaves entirely glabrous while shaded parts of the plants contained branches with leaves pubescent mid ribs on the undersides. This was most distinctly observed on the branches and midrib of the underside of the leaves of *Costion 3433,* which includes cuttings from a new growth stem (pubescent) and a mature stem (glabrous). Collection *Costion 3359* is exceptional in also showing the lower leaves in the shade to be more than three times the length and width of the leaves higher up in the canopy. This phenomenon was even observed in shorter shrubby plants in open exposed savanna surrounded by waist high *Dicranopteris* ferns (*Costion 3364*). The branches shaded by the *Dicranopteris* contained the diagnostic slightly pubescent midrib on the underside whereas the branches above the ferns were glabrous. In addition three specimens from saplings were collected (*Costion 3372, 3373, and 3374*) which were all densely pubescent in their juvenile stage. Fosberg and Sachet (1987) proposed a variety of *T. subauritus, T. subauritus* var. *strigosus*, distinguished only by its pubescent new growth, leave veins, and petioles. Their description was based only on three cited specimens and did not include any ecological information about the specimens. Since the recent observations documented here clearly show specimens with and without these characters on the same plants this variety is not well supported. The characters used to distinguish *T. subauritus* var. *strigosus* are better explained by a different growth stage and/or adaptations of the plants to localized ecological factors.

SPECIMENS EXAMINED: PALAU: Babeldaob: Canfield 355, 588, 593, 594, 601, 695B, 772 (US, BISH); Eastlick LR 23315 (US); Fisher 115, 123, 127a, 127b (US); Fosberg 32573, 47681, 50593 (US); Herbst 9389a, 9438, 9444 (US, BISH); Hosoakawa 3362, 3416 (US, BISH); Kanehira 505, 1962, 1932, 2069, 2323, 2344, 1928, 4463, 4499 (FU); Kanehira 145 (FU, TOFO); Kanehira 2284 (FU, US); Otobed 152 (US); Raulerson 5569, 6051, 16845 (US); Costion 3359, 3362, 3363, 3364, 3365, 3366, 3370, 3372, 3373, 3374, 3431, 3432, 3433, 3435, 3436, 3437 (CNS, BNM); Rinehart LR 26449 (BISH); Malakal: Costion 3268, 3271, 3272, 3273, 3334, 3377, 3378, 3379, 3494, 3495, 3496 (CNS, BNM); Ngarekebesang: Costion 3501 (CNS, BNM).

**Data limitations and unresolved taxonomy**

The primary limitation of this study was the inability to identify any material of *Timonius corymbosus* var. corymbosus additional to the Lectotype in herbaria or the field even after extensive surveys across the archipelago. An additional complication is that *T. corymbosus* is only known from a male specimen with a staminate inflorescence, which is more difficult to distinguish than a mature female specimen with fruits. Although it would have increased the accuracy of our results, not including this taxon does not change the conservation implications found here for *T. salsedoi,* which was the primary aim of this study. Further work that can extract DNA from the type of *Timonius corymbosus* or identify new fertile female specimens in the field that match the type is deemed critical for a complete revision of all *Timonius* species in the Palau and the larger Micronesia bioregion.
