# Supplementary material for: DNA Barcodes Confirm the Taxonomic and Conservation Status of a Species of Tree on the Brink of Extinction in the Pacific
Source: PLoS One. 2016 Jun 15;11(6):e0155118. doi: 10.1371/journal.pone.0155118 (PMC4909193; doi:10.1371/journal.pone.0155118)
Supplement: S2 Table — List of PCR mixtures and thermal profiles used for each locus. (DOCX) [file pone.0155118.s002.docx]

| **Locus** | **PCR mixture** | **PCR thermal profile** |
| --- | --- | --- |
| *rbcLa* | 20uL reaction volume containing 1U Kapa Taq polymerase with final concentrations of 1.25x PCR Buffer (Invitrogen 10x PCR buffer without MgCl_2_), 3mM MgCl_2_, 0.25mM dNTP’s, 0.6μM each primer, 0.05mg/ml BSA and 4% DMSO. Genomic DNA (not quantified) was added at 2ul per reaction. | 95° for 90s; 35 cycles of 95° for 30s, 50° for 1min, 72° for 90s; final incubation at 72° for 10min. |
| *matK* | 20uL reaction volume containing 1U Kapa Taq polymerase with final concentrations of 1.25x PCR Buffer (Invitrogen 10x PCR buffer without MgCl_2_), 3.125mM MgCl_2_, 0.25mM dNTP’s, 0.3μM each primer, 0.08mg/ml BSA and 4% DMSO. Genomic DNA (not quantified) was added at 2ul per reaction. | 95° for 2min; 10 cycles of 95° for 30s, 56° for 20s, 72° for 50s; 25 cycles of 88° for 30s, 56° for 20s, 72° for 50s; final incubation at 72° for 5min. |
| *trnH–psbA* | 20uL reaction volume containing 0.5U iProof™ polymerase with a final concentration of 1x PCR Buffer (iProof 5x HF Buffer containing 7.5mM MgCl_2_*), 0.2mM dNTP’s, 0.1μM each primer and 3% DMSO. Genomic DNA (not quantified) was added at 2ul per reaction. | 98° for 45s; 35 cycles of 98° for 10s, 64° for 30s, 72° for 40s; final incubation at 72° for 10min. |
| *ITS* | 20uL reaction volume containing 1U Kapa Taq polymerase with final concentrations of 1.25x PCR Buffer (Invitrogen 10x PCR buffer without MgCl_2_), 1.875mM MgCl_2_, 0.25mM dNTP’s, 0.3μM each primer, 0.05mg/ml BSA and 4% DMSO. Genomic DNA (not quantified) was added at 2ul per reaction. | 97° for 5min; 35 cycles of 97° for 30s, 55° for 1min, 72° for 2min; final incubation at 72° for 10min. |
| *trnL-trnF* | 20uL reaction volume containing 0.5U iProof™polymerase with a final concentration of 1x PCR Buffer (iProof 5x HF Buffer containing 7.5mM MgCl_2_*), 0.2mM dNTP’s, 0.4μM each primer and 3% DMSO. Genomic DNA (not quantified) was added at 2ul per reaction. | 98° for 45s; 35 cycles of 98° for 10s, 65° for 30s, 72° for 45s; final incubation at 72° for 10min |
| *atpB-rbcL* | 20uL reaction volume containing 0.75U Kapa Taq polymerase with a final concentration of 1x PCR Buffer (KAPA 10x PCR Buffer A containing 15mM MgCl_2_*), 0.2mM dNTP’s, 0.4μM each primer. Genomic DNA (not quantified) was added at 2ul per reaction. | 95° for 4min; 35 cycles of 95° for 30s, 50° for 1min, 72° for 1min; final incubation at 72° for 5min |
| *petD* | 20uL reaction volume containing 1U Kapa Taq polymerase with a final concentration of 1x PCR Buffer (KAPA 10x PCR Buffer B containing 15mM MgCl_2_), additional MgCl_2_ to a final reaction concentration of 2.75mM MgCl_2_, 0.25mM dNTP’s, 0.2μM each primer and 4% DMSO. Genomic DNA (not quantified) was added at 2ul per reaction. | 96° for 90s; 33 cycles of 95° for 30s, 50° for 1min, 72° for 90s; final incubation at 72° for 10min |

* Gives a final reaction concentration of 1.5mM MgCl_2_
